# Using Natural Language Processing to Explore Mental Health Insights From UK Tweets During the COVID-19 Pandemic: Infodemiology Study

**DOI:** 10.2196/32449

**Published:** 2022-03-31

**Authors:** Christopher Marshall, Kate Lanyi, Rhiannon Green, Georgina C Wilkins, Fiona Pearson, Dawn Craig

**Affiliations:** 1 National Institute for Health Research Innovation Observatory Newcastle University Newcastle United Kingdom

**Keywords:** Twitter, mental health, COVID-19, sentiment, lockdown, soft intelligence, artificial intelligence, machine learning, natural language processing

## Abstract

**Background:**

There is need to consider the value of soft intelligence, leveraged using accessible natural language processing (NLP) tools, as a source of analyzed evidence to support public health research outputs and decision-making.

**Objective:**

The aim of this study was to explore the value of soft intelligence analyzed using NLP. As a case study, we selected and used a commercially available NLP platform to identify, collect, and interrogate a large collection of UK tweets relating to mental health during the COVID-19 pandemic.

**Methods:**

A search strategy comprised of a list of terms related to mental health, COVID-19, and lockdown restrictions was developed to prospectively collate relevant tweets via Twitter’s advanced search application programming interface over a 24-week period. We deployed a readily and commercially available NLP platform to explore tweet frequency and sentiment across the United Kingdom and identify key topics of discussion. A series of keyword filters were used to clean the initial data retrieved and also set up to track specific mental health problems. All collated tweets were anonymized.

**Results:**

We identified and analyzed 286,902 tweets posted from UK user accounts from July 23, 2020 to January 6, 2021. The average sentiment score was 50%, suggesting overall neutral sentiment across all tweets over the study period. Major fluctuations in volume (between 12,622 and 51,340) and sentiment (between 25% and 49%) appeared to coincide with key changes to any local and/or national social distancing measures. Tweets around mental health were polarizing, discussed with both positive and negative sentiment. Key topics of consistent discussion over the study period included the impact of the pandemic on people’s mental health (both positively and negatively), fear and anxiety over lockdowns, and anger and mistrust toward the government.

**Conclusions:**

Using an NLP platform, we were able to rapidly mine and analyze emerging health-related insights from UK tweets into how the pandemic may be impacting people’s mental health and well-being. This type of real-time analyzed evidence could act as a useful intelligence source that agencies, local leaders, and health care decision makers can potentially draw from, particularly during a health crisis.

## Introduction

COVID-19 was identified as a new type of coronavirus in early January 2020 [1]. Since then, the disease has rapidly spread to and affected almost all parts of the world. In the United Kingdom, the first outbreak was reported on January 31, 2020, with a national lockdown following on March 26, 2020. Shortly before this, COVID-19 was declared a global pandemic by the World Health Organization (WHO) on March 11, 2020 [2,3].

The COVID-19 pandemic continues to have a profound effect on mental health [4]. In a key position paper published in June 2020, the authors explored the current and future potential psychological, social, and neuroscientific effects of COVID-19 and set out a series of priorities and longer-term strategies for mental health research [4]. One of the immediate research priorities presented in the paper was “surveillance.” In particular, the authors suggested that finding useful ways to monitor and analyze data on the mental health effects of the COVID-19 pandemic across the whole population, as well as vulnerable subgroups, was essential [4].

With over 300 million active monthly users, Twitter is one of the most popular social media platforms available. Twitter is a free microblogging service that enables its users to post, read, and respond to each other’s “tweets” (ie, short messages limited to 280 characters). Social media data are being increasingly used as a data source to inform health-related research, with the potential for offering a more efficient means of data collection over traditional, time-consuming, and costly survey-based methods [5]. In particular, Twitter has been used to monitor, track trends, and disseminate health information during past viral pandemics [6-9]. Further, previous studies have successfully leveraged Twitter data for the assessment of public sentiments, attitudes, and opinions concerning health-related issues [10,11].

Channels of soft intelligence like Twitter, leveraged using novel artificial intelligence (AI) techniques (including natural language processing [NLP]), offer an opportunity for real-time analysis of public attitudes, sentiments, and key topics of discussion [12]. As aforementioned, previous case studies have shown that applying NLP can aid health researchers in gaining insights from large, unstructured data sets, such as Twitter. However, the true value of this type of work, including the data set itself, analysis methods, and how it might be integrated into more formal public health research outputs, is still uncertain. For example, a lot of previous work so far has focused on the use of internally developed, bespoke tools or packages, which tend to require a certain level of technical expertise around machine learning (ML) in order operate effectively. However, as methods continue to mature, we are seeing a growing number of “off-the-shelf” solutions become available, which appear to be more accessible and require less technical understanding of the underlying ML concepts.

The aim of this study was to further explore the value of soft intelligence as a meaningful source of evidence, which, when analyzed using an accessible NLP platform, can support public health research activity. In this article, we report the findings from a case study that examined a large collection of tweets relating to mental health posted from the United Kingdom during the COVID-19 pandemic.

## Methods

### Data Collection

An advanced AI-based, text analytics platform using NLP was used to initially analyze the tweets. The analytics platform, “Wordnerds,” is described by its developers as a “text analysis and insights platform using machine learning techniques” [13]. In particular, this off-the-shelf platform supports analysis of metadata, topic, and sentiment to understand the context of a tweet and to group tweets together into topic clusters that contain tweets relating to each other or discussing similar issues. This facilitates a more accurate and sophisticated insight into the vaccine conversation on Twitter compared with methodologies that rely solely on a qualitative count of single words, phrases, or hashtags [14].

We developed a search strategy comprising a list of terms related to COVID-19, the lockdown, and mental health to search (or “scrape”) for relevant tweets (see Textbox 1).

**Search strategy for relevant tweets.**

Corona OR covid OR lockdown

AND

mental health OR anxiety OR depression OR anxious OR depressed OR depressing OR trauma OR traumatic OR “obsessive compulsive disorder” OR OCD OR vulnerable OR loneliness OR lonely OR isolated OR isolation OR sleep OR stress OR stressful OR self-harm OR self-harming OR suicide OR suicidal OR well-being

Search terms were identified through discussion within the research team and scanning recent literature around mental health. Once the strategy had been agreed upon, it was reviewed by a topic expert and information specialist. We then began prospectively searching for and scraping relevant tweets using Twitter’s advanced search application interface [15]. A geolocation filter was applied to the search strategy to limit the collection of tweets to those posted in the United Kingdom only.

In this article, we report the findings from our analysis of relevant tweets in the United Kingdom collected over a 24-week period, from July 23, 2020 to January 6, 2021.

### Preparing and Cleaning the Data

All collated tweets were anonymized. Before analyzing the data, the retrieved results were run through a final keyword filter. This filter was comprised of a series of terms and keywords associated with mental health problems to help ensure a more relevant, cleaner, and less noisy data set for analysis. For example, general terms such as “isolation” and “well-being” were filtered out. Further, terms associated with eating disorders were added alongside the original terms.

The final keyword filter applied is summarized in Textbox 2.

**Keyword filter used to clean the data set.**

mood OR “mental health” OR depression OR anxiety OR anxious OR depressed OR depressing OR trauma OR traumatic OR OCD OR compulsive OR vulnerable OR loneliness OR lonely OR isolated OR sleep OR stress OR stressful OR self-harm OR self-harming OR suicide OR suicidal OR anorexia OR anorexic OR bulimia OR bulimic OR eating disorder OR binge eating OR OFSED (other specified feeding or eating disorder)

### Data Analysis

We used Wordnerds to interrogate the tweets. The developers state that their platform uses a range of different technologies in order to deliver its various analyses, including contextual word embeddings and collocation methods [13]. To date, we have found no other published studies coordinated by an academic research group that have used this specific tool.

Using the platform, we were able to track and determine the weekly frequency of tweets relevant to our initial search strategy from the United Kingdom between July 23, 2020 and January 6, 2021. We also tracked the frequency of subsets of these tweets that incorporated terms for specific mental health problems, as listed in Textbox 3.

The NLP platform was then used to explore the sentiment (ie, positive, neutral, or negative) of the whole corpus of tweets. Sentiment was determined using contextual word embedding techniques, including classification of grammar to understand how words interact [16].

**Keyword filters used to identify specific mental health problems.**

**Anxiety:** “anxious,” “anxiety”

**Depression:** “depression,” “depressed,” “depressing”

**Stress:** “stress,” “stressful,” “stressed”

**Loneliness:** “loneliness,” “lonely,” “alone”

Following sentiment analysis, the platform’s topic analysis feature was used to identify and cluster key emerging topics of discussion, both with positive and negative underlying sentiment. For this analysis, the platform automatically clustered key topics of positive and negative discussion using topic collocation methods. This is a probabilistic method of identifying interesting sentence fragments and words that occur frequently together within a data set. The results of the platform’s topic analysis were examined, and its findings were summarized by 2 of the authors (KL and RG). These summaries were checked by 2 further authors (CM and GCW).

Due to the high volume of tweets collected, the topic analysis was split between 2 equal time periods. The first covered summer 2020 to autumn 2020, when lockdown restrictions were relaxed. The second covered the autumn to winter period in 2020, when regional and then further national lockdown restrictions were introduced.

### Ethical Considerations

Institutional review board approval was not sought as this study used only publicly available data. All posts were de-identified, and there was no direct interaction with Twitter users.

## Results

### Tweet Volume

**Figure 1:**
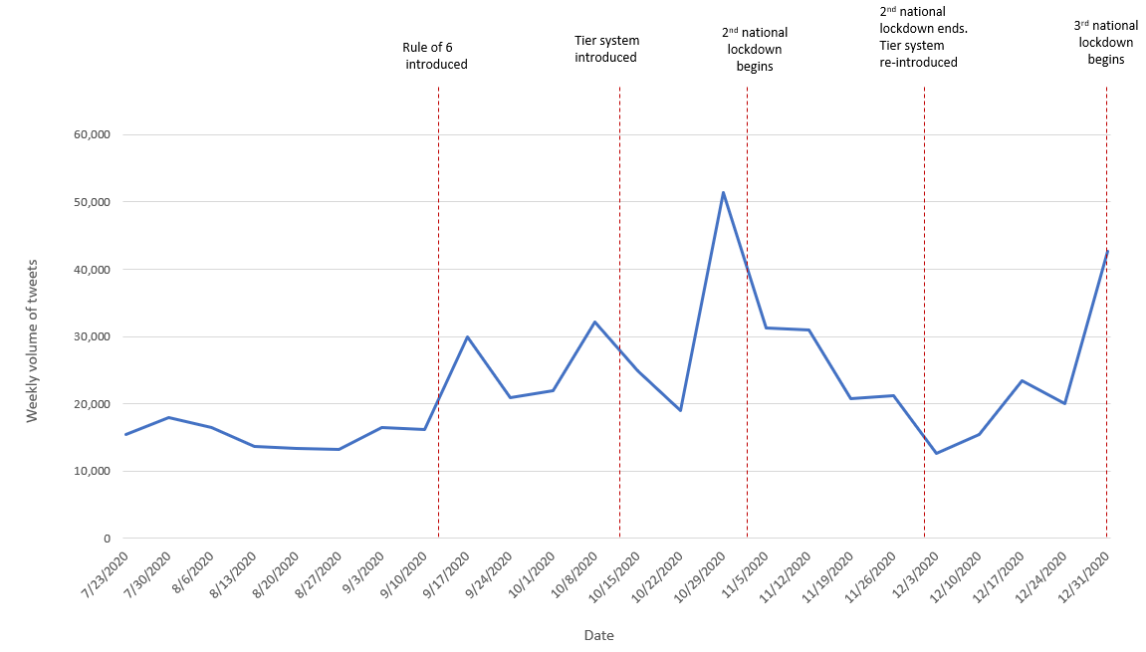
Volume of tweets from July 23, 2020 to January 6, 2021. Key events during the study period included the (1) rule of 6 (up to 6 people from any number of households could meet indoors or outdoors), (2) tier system (regions across England were assigned a tier from 1 to 3 based on epidemiological indicators, and these tiers dictated the restrictions in that area, such as which businesses could open and how many individuals could meet in a group during national lockdown—nonessential businesses were closed and people were prohibited from meeting outside of their support bubble).

We captured and collated 286,902 tweets posted by users in the United Kingdom from July 23, 2020 to January 6, 2021. The volume of tweets by week, together with key events taking place during this study period, is visualized in Figure 1. Further notable events or issues that occurred throughout the study period are summarized in Table 1 (weeks 1 to 12) and Table 2 (weeks 13 to 24).

As shown in Figure 1, the highest volume of tweets occurred week commencing (w/c) October 29, 2020, with 51,340 tweets. The lowest volume was observed w/c December 3, 2020, with 12,622 tweets. The data show a fairly consistent baseline trend over the study period. Spikes in the volume of tweets occurred in September, October, and December, typically during periods leading up to (or during) a major change in social distancing and lockdown measures across the United Kingdom.

The first peak was observed w/c September 17, 2020, the week after the introduction of the “rule of 6,” whereby a mix of 6 people from any household could meet indoors or outdoors. A similar peak was observed w/c October 8, 2020. This was the week leading up to the government’s introduction of a new tiered system, whereby regions across the United Kingdom were allocated to 1 of 3 tiers (and later a fourth tier) based on prevalence of COVID-19. Higher tiers corresponded with tighter restrictions, including closing nonessential businesses and limits placed on social gatherings.

The largest peak was observed w/c October 29, 2020, the week before the second national lockdown began. The final peak occurred w/c December 31, 2020, the week leading up to the start of a third national lockdown. For all 3 national lockdowns, all nonessential businesses were closed, and UK residents were restricted from meeting anyone outside of their “social bubble” (ie, their household or, for people living alone, themselves plus one other household).

**Table 1:** Notable events that occurred from July 23, 2020 to October 14, 2020.

Weeks	Events
Weeks 1-2	“Health Protection Regulations 2020” comes into force (ie, mandatory wearing of face masks in most indoor establishments). Gyms, swimming pools, and other indoor sports facilities reopen. England reports highest number of excess deaths in Europe for February to June. Major incident is declared in Greater Manchester after rise in cases.
Weeks 3-4	Stricter measures are reintroduced in Preston, Lancashire. England’s revamped contact-tracing app begins public trials. General Certificate of Secondary Education (GCSE) results are published with grades based on teachers’ assessments. The Education Secretary confirms free appeals for A-Level and GCSE.
Weeks 5-6	Ban on property evictions is extended until September 20. Greater Manchester Police report breaking up 126 illegal gatherings. Thousands of lockdown protesters gather in Trafalgar Square.
Weeks 7-8	Health experts express doubt about mass testing plan: “Operation Moonshot.” World Suicide Prevention Day is on September 10. “Rule of 6” for indoor and outdoor gatherings is announced.
Weeks 9-10	Local lockdown measures are announced for Newcastle. Restrictions are relaxed for childcare purposes between households. Couples in established relationships can meet without social distancing. Second version of National Health Service (NHS) contact-tracing app becomes publicly available. £10,000 (US $13,035) fine for failing to self-isolate is announced.
Weeks 11-12	Lockdown restrictions are tightened in the Northeast. Tier system comes into force, replacing local lockdowns. World Mental Health Day occurs on October 10.

**Table 2:** Notable events that occurred from October 15, 2020 to January 6, 2021

Weeks	Events
Weeks 13-14	Shielding ends for clinically vulnerable Additional financial support to businesses in Tier 3 is announced.
Weeks 15-16	Month-long national lockdown in England from November 5, 2020 is announced. Clinically vulnerable are asked to shield again. Furlough scheme is extended until March 2021. Pfizer/BioNTech press release announces vaccine is 90% effective. Travel window for university students to return for Christmas is announced.
Weeks 17-18	Government publishes their “staying mentally well this winter” guidance. Plans to ease restrictions for 5 days over Christmas are announced.
Weeks 19-20	Indoor care home visits can resume subject to lateral flow test. Medicines and Healthcare products Regulatory Agency (MHRA) approves Pfizer/BioNTech vaccine for rollout in the United Kingdom. Joint Committee on Vaccination and Immunisation (JCVI) publishes vaccine priority groups. National lockdown ends, and Tier system resumes. Most regions in England are placed in Tier 2 or 3. Shielding ends for clinically vulnerable. First COVID-19 vaccine is administered.
Weeks 21-22	Self-isolation period is reduced from 14 days to 10 days. New variant of the virus is identified. Further regions of the United Kingdom enter highest Tier. Furlough scheme is extended to April 2021. Large parts of Southeast England move into the new, stricter Tier 4. Christmas relaxation period is reduced from 5 days to 1 day. Travel restrictions for South Africa are enforced. Most of England will enter Tier 4 from December 26, 2020 is announced.
Weeks 23-24	MHRA approves AstraZeneca/Oxford vaccine. JCVI publishes updated guidance. UK Prime Minister announces third national lockdown from January 4, 2021, with schools remaining closed.

### Mental Health Problems

Table 3 summarizes the volume of tweets that utilized at least one of the terms related to anxiety, depression, stress, or loneliness. In total, 113,312 (39.50%) of the 286,902 tweets scraped through the initial search strategy related to anxiety, depression, stress, or loneliness.

**Figure 2:**
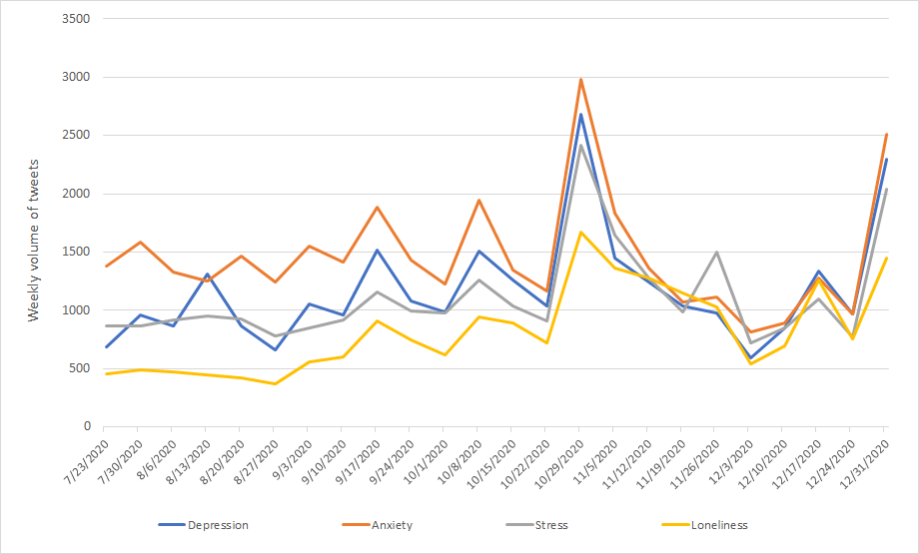
Volume of tweets from July 23, 2020 to January 6, 2021.

Figure 2 presents the volume of tweets utilizing terms related to anxiety, depression, stress, or loneliness around each keyword filter over the study period.

Across all of the mental health problems that were focused on here, the highest volume of tweets was observed w/c October 29, 2020, and the lowest volume of tweets occurred w/c December 3, 2020. The “Anxiety” filter returned the highest total number of tweets, and the “Loneliness” filter returned the lowest (see Table 3). The trend in tweet frequency for each filter mirrored the trend reported for the overall data set over the study period.

During the first half of the analysis period (w/c July 23, 2020 to w/c October 22, 2020), tweet volume between the 4 mental health problem filters varied. In particular, tweets related to the “Anxiety” filter were consistently posted most often, and tweets relating to the “Loneliness” filter were consistently posted least. There was a spike in volume across all 4 filters w/c October 29, 2020, the start of the second national lockdown. Following the spike, the volume of tweets across all of the filters was broadly similar for the remainder of the analysis period.

**Table 3:** Keyword filters and resultant volume of tweets for specific mental health problems.

Filter	Keyword terms	Tweets (N=113,312)
Anxiety	“anxious,” “anxiety”	37,213 (32.84%)
Depression	“depression,” “depressed,” “depressing”	29,523 (26.05%)
Stress	“stress,” “stressful,” “stressed”	26,725 (23.59%)
Loneliness	“loneliness,” “lonely,” “alone”	19,851 (17.52%)

### Sentiment Analysis

Of the 286,902 tweets, 34,347 (11.97%) were identified as having positive sentiment, 217,728 (75.89%) as having neutral sentiment, and 34,827 (12.14%) as having negative sentiment, with an overall sentiment score of 50% assigned. A score below 50% suggests negative sentiment, and a score greater than 50% suggests positive sentiment. Textbox 4 presents a selection of example tweets that the NLP platform classified as both positive and negative.

**Figure 3:**
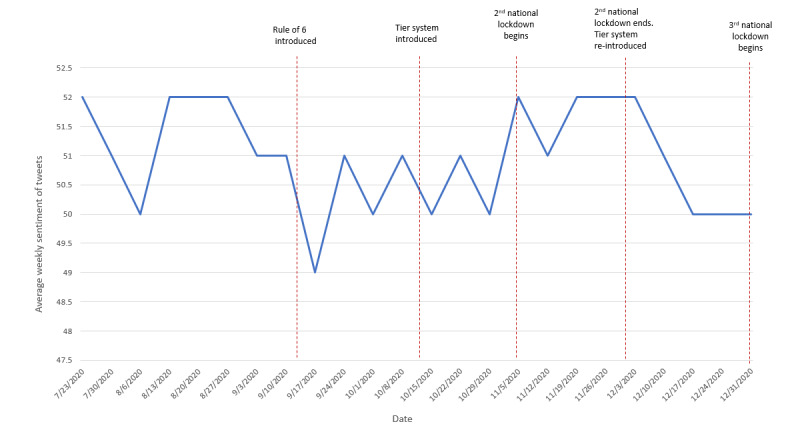
Sentiment of tweets from July 23, 2020 to January 6, 2021.

Here, the overall score of 50% indicates neutral sentiment across all tweets over the study period. Figure 3 visualizes the weekly change in sentiment over the study period from July 23, 2020 to January 6, 2021.

**Examples of positive and negative tweets classified by the NLP platform.**

**Example tweets classified with positive sentiment**

...Since lockdown my anxiety has dramatically gotten so much better. I used to get stress spots, panic attacks before presentations, etc. Na bruh, working from home has been a life saver...

If anyone is struggling with lockdown (or wants to reduce stress/anxiety). I would really recommend trying gratitude journaling. I’ve shared some tips below – I hope they’ll be helpful

…Never been so happy to set foot in a gym today. I feel so much happier getting back into a routine and pleased to see I haven’t lost much strength through pregnancy and lockdown. It’s made me realise how important it is for my own mental health...

**Example tweets classified with negative sentiment**

...I’m finding this lockdown harder than the last one. For me it's not just the weather, it’s having no end in sight which is a struggle when you suffer with anxiety and depression...

…genuinely think a second lockdown would completely destroy my mental health. I’m nervous about the next few weeks/months...

Feeling absolute rubbish today, feel like my mental health is debilitating can’t bring myself to get out of bed, my only escape is drinking with to forget these awful times

Sentiment remained neutral or positive throughout most of the study period. The highest assigned sentiment score, 52%, occurred in weeks 1, 4 to 6, 16, and 18 to 20. The lowest assigned sentiment score, 49%, occurred in week 9 (w/c September 17, 2020).

Overall, the data show a relatively consistent trend in sentiment over the study period. When sentiment fluctuation did occur, it was similar to the trend observed with tweet frequency and coincided with major changes to lockdown or social distancing rules.

### Topic Analysis

In this section, results are reported for 2 time periods, as follows: (1) July 23, 2020 to October 14, 2020 and (2) October 15, 2020 to January 6, 2021.

#### Results of the Topic Analysis for Weeks 1 to 12

This section presents the results of a topic analysis based on 115,700 scraped tweets posted from July 23, 2020 to October 14, 2020.

#### Summary of Clustered Topics With Positive Sentiment

“Mental health” emerged as a key topic of discussion underpinned with positive sentiment. The importance of mental health throughout the “coronavirus pandemic,” as a critical health issue, was shared widely by people on Twitter. During this period, people openly discussed their mental health and how they had been coping. People also shared praise for specific local and national mental health services, as well as key public figures (eg, Marcus Rashford).

There was considerable discussion based around “World Mental Health Day” and “World Suicide Prevention Day.” People were sharing helpful strategies (eg, videos, charities, help lines, exercise regimes, healthy eating advice) others could use to protect and maintain their mental health. There were also calls from people to be particularly vigilant and make sure they are checking in with any “vulnerable people” in their life.

Positive discussion was observed around “working from home.” Various users were sharing helpful resources to support working from home effectively, including strategies that people had found useful during the previous national lockdown. Some people reported that mandatory working from home had helped them to achieve a better work-life balance and reduced their anxiety.

Specific examples of mental health tweets underpinned with positive sentiment from weeks 1 to 12 are presented in Textbox 5.

**Sample of clustered tweets from weeks 1 to 12 with positive sentiment.**

...Never been so happy to set foot in a gym today. I feel so much happier getting back into a routine and pleased to see I haven’t lost much strength through pregnancy and lockdown. It’s made me realise how important it is for my own mental health...

...I eventually took time out from March as it just got too much. My anxiety was through the roof and frankly I was glad of lockdown - I didn’t want to leave the house anyway. I’m in a much better place now thankfully...

...This coming Thursday is world suicide prevention day and we need to be talking about this in the context of covid, lockdown, the economy and more. Most of all we need to talk and respond with compassion and support...

...As many return to working from home, worth reminding ourselves of all those self-care tips we were bombarded with in March. @[redacted] on managing anxiety and emotional fatigue...

...Since lockdown my anxiety has dramatically gotten so much better. I used to get stress spots, panic attacks before presentations, etc. Na bruh, working from home has been a life saver...

...Lockdown was great for my mental health despite not being furloughed. While working from home I experienced being able to sleep well, eat well, exercise regularly, hear myself think, and feel alive, rather than dragging myself through each day merely surviving to begin the next...

#### Summary of Clustered Topics With Negative Sentiment

“Second lockdown” emerged as a key topic of discussion underpinned with negative sentiment. People were sharing their fears, concerns, and anxieties over the prospect of a second national lockdown and the impact this would have on theirs (and other’s) mental health. People recalled and spoke openly about how their mental health had suffered during the previous lockdown, referencing specific problems such as “anxiety,” “depression,” and posttraumatic stress disorder. Some people shared that they had been diagnosed with depression for the first time due to the previous national lockdown.

Many people were angry that not enough had been done by the government to protect “vulnerable people” during the previous national lockdown. Suicide was also discussed. People were claiming that suicide rates had increased during lockdown, particularly among younger people. There was widespread sharing of warnings from key educational figures that the pandemic would have long-lasting negative effects on “children.” Further, some argued that people were using “mental health” as an excuse to avoid further lockdown restrictions. Many people were concerned that a second lockdown would be much worse for people’s mental health than the first (the lockdown coinciding with winter and students returning to university were both seen as contributing negative factors). Those who refused to wear masks were a further source of anxiety for some people, with a high proportion of tweets calling on others to “wear a mask.” People also discussed COVID-19 tests during this period. In particular, some people shared how stressful and anxiety-inducing taking the test, and also waiting for the results, can be.

Tweets contained within some of the other topic clusters generated by the platform, such as “care homes” and “covid deaths,” did not appear to be related to mental health.

Specific examples of mental health tweets underpinned with negative sentiment from weeks 1 to 12 are presented in Textbox 6.

**Sample of clustered tweets from weeks 1 to 12 with negative sentiment" to be consistent with the other textboxes.**

...went into the year hopeful and ready to turn my life around...then covid happened and the world became a giant prison. Depression slowly came back, anxiety eating away and all emotion and feeling slowly dry up. Mind became a wasteland pretty much. Lifeless and desert...

...I have to get a COVID test done before going back to work at the end of the month and the anxiety I am having from the thought of them sticking that thing up my nose...

...I’m finding this lockdown harder than the last one. For me it's not just the weather, it’s having no end in sight which is a struggle when you suffer with anxiety and depression...

...genuinely think a second lockdown would completely destroy my mental health. I’m nervous about the next few weeks/months...

...I'm all for people following the rules - wear your masks and keep distance when required, but this is very true and will make people more depressed by lack of communication with real people. Depression will be a huge and sad factor if this new lockdown does go ahead. Stay safe...

...This bizarre competition between suicide and Covid deaths needs to end. I understand the mental health issues that can arise from lack of social interaction and other factors from Covid but this weird death competition between the 2 needs to stop...

#### Results of the Topic Analysis for Weeks 13 to 24

This section presents the results of a topic analysis of 171,202 scraped tweets posted from October 15, 2020 to January 6, 2021.

Table 2 summarizes a selection of notable events that occurred during this time period.

Tables 4 and 5 present the top 10 most discussed topics that occurred throughout the study period.

**Table 4:** Top 10 most discussed positive and negative topics from July 23, 2020 to October 14, 2020.

Ranking			Clustered topic		Number of tweets
**Topics with positive sentiment**					
	1	“mental health”		5499	
	2	“vulnerable people”		1513	
	3	“lockdown anxiety”		491	
	4	“communities... support”		348	
	5	“health... important”		327	
	6	“coronavirus pandemic”		266	
	7	“work from home”		257	
	8	“support... provide”		246	
	9	“improved... health”		234	
	10	“world day”		233	
**Topics with negative sentiment**					
	1	“mental health”		5192	
	2	“people... vulnerable”		1421	
	3	“children... coronavirus”		1037	
	4	“covid... deaths”		743	
	5	“wear a mask”		566	
	6	“test for covid”		419	
	7	“lockdown... gone”		411	
	8	“care homes”		405	
	9	“second lockdown”		377	
	10	“anxiety... depression”		365	

**Table 5:** Top 10 most discussed positive and negative topics from October 15, 2020 to January 6, 2020..

Ranking		Clustered topic	Number of tweets
**Topics with positive sentiment**			
	1	“suicide lockdown”	10,096
	2	“mental health”	10,031
	3	“people…vulnerable”	1860
	4	“friends or family”	526
	5	“need…support”	451
	6	“ones you love”	409
	7	“community…helping”	306
	8	“stress & anxiety	295
	9	“night’s sleep”	275
	10	“difficult times”	259
**Topics with negative sentiment**			
	1	“mental health“	10,299
	2	“vulnerable people”	1767
	3	“going…lockdown”	1025
	4	“deaths…covid”	979
	5	“suicide rates”	490
	6	“sleep at night”	479
	7	“lockdown…depressing”	474
	8	“committed suicide”	460
	9	“coronavirus…children”	441
	10	“wearing masks”	441

#### Summary of Clustered Topics With Positive Sentiment

During this period, a tweet suggesting suicide rates had risen by 200% since lockdown was shared widely. The tweet contained contact details for a registered UK charity, Samaritans, urging people to reach out for support if needed. This viral tweet resulted in our analysis platform recognizing “suicide lockdown” as a key topic of discussion. The information being reported by this tweet was not accurate [17,18].

As with the previous 12 weeks, “mental health” remained a key topic of discussion underpinned with positive sentiment during this period. People discussed how the lockdown had, in some ways, had a positive impact on their mental health. Various people and organizations continued to share practical tips on how to support one’s mental health, particularly around strategies to reduce “stress and anxiety.”

Many people continued to express concern for the mental health of perceived “vulnerable people” during lockdown, including children, young people, disabled people, those with learning difficulties, and those with any pre-existing mental health problems. Some people were calling on the government to provide further support for these groups as lockdown restrictions tightened. Many people were encouraging those that that were struggling to stay connected with others and reach out to “ones you love” and “friends and family.”

“Sleep” emerged as a key topic of discussion with positive sentiment. Many people discussed how important getting a good “night’s sleep” was for their mental health, particularly during these “difficult times.” People shared relaxation techniques they had used, which had helped them to fall asleep, and which may help others too.

Specific examples of mental health tweets underpinned with positive sentiment from weeks 13 to 24 are presented in Textbox 7.

**Sample of clustered tweets from weeks 13 to 24 with positive sentiment.**

Suicide figures are up 200% since lockdown. Could two followers please copy and re-post this tweet? We’re trying to demonstrate that someone is always listening. Call 116 123 (Samaritans UK). Just two. Any two. Copy, not RT. #MentalHealth #SuicidePrevention #SuicideAwareness

important to remember with all the 200% increase in suicide tweets. Raising awareness is fantastic, but make sure it’s factual. #SuicidePrevention.

My mental health is sooo much better now it’s a real lockdown again. However I know this isn’t a universal experience. For those who find lockdown harder for whatever reason, I’m a) sending virtual hugs if wanted but b) reminding you it’s STRONG to reach out to a helpline!

As we start lockdown 3, a reminder that your situation does not have to be the worst for it to suck and for you to get help! Reach out to your loved ones & professional mental health support if you need it. Stay safe.

Such a tough time at the moment for everyone, another lockdown especially in winter can be devastating for mental health. My dm’s are always open for anyone who needs a chat – be kind and check on your loved ones.

Thanks to local organisations, community groups and faith institutions that have provided vital services, human support and companionship, in person and online, to many vulnerable people during #Covid-19. We will keep working with you all to build stronger and united communities.

If anyone is struggling with lockdown (or wants to reduce stress/anxiety). I would really recommend trying gratitude journaling. I’ve shared some tips below – I hope they’ll be helpful

#COVID19 wellbeing tip: make sure you get a good night’s sleep! A good rest is so important for your mental and physical health, managing stress and much more. If you’re struggling with sleep, try these tips and check out our Sleep self-help guide.

#### Summary of Clustered Topics With Negative Sentiment

“Mental health” continued to be widely discussed during this final 12-week period. Many people reflected on the negative impact that lockdowns had had on their mental health. There were concerns from some that any progress they had made with their mental health would be lost with another lockdown. There was continued anger toward the UK government about a perceived lack of support for those struggling with their mental health.

There was a lot of discussion around the looming national lockdown announced for January 4, 2021. Many people expressed concern about how long this lockdown would last and their hope that this would be the final lockdown. Some shared that they would be defying restrictions in order to prioritize their mental health. Others continued to argue that people were using their mental health as an excuse for not following the rules. There was continued worry about how the lockdown would affect perceived “vulnerable people.”

During this period, people shared their thoughts about the vaccine rollout. In particular, people were concerned about the length of time between jabs and the number of canceled vaccination appointments being reported by the media.

“Sleep” continued to be a key topic of discussion with many people sharing how they had not been sleeping well. Some people shared how they had been increasing their alcohol intake in an effort to help them sleep.

Specific examples of mental health tweets underpinned with negative sentiment from weeks 13 to 24 are presented in Textbox 8.

**Sample of clustered tweets from weeks 13 to 24 with negative sentiment.**

Well lockdown 3.0 has barely started and I can already feel all the hard work I put balancing my mental health slip away

Tbh I just hope my mental health doesn’t become as bad as first lockdown

Feeling absolute rubbish today, feel like my mental health is debilitating can’t bring myself to get out of bed, my only escape is drinking with to forget these awful times

There needs to be more mental health support @BorisJohnson as that will be one of the highest collateral costs of this. People like me are struggling badly in isolation and with mental health issues and there isn’t enough support. #Uklockdown #Covid

My mental health is already rock bottom due to not seeing my family/friends, being overworked and not sleeping – god help what another lockdown is gonna do to me

… everyone is talking about lockdown 3 but carehomes haven’t left lockdown since the first lockdown started. Everyone is ignoring how vulnerable people feel, disables people or careworkers and carers.

...I’ve never had ocd but do have A LOT of anxiety, + have noticed the longer lockdown goes on the more my anxious behaviours start to look like obsessive ones eg. I check my cooker before bed/before leaving my house when I never did that pre lockdown, also started counting.!

It’s only our second day of lockdown 3.0 going solo & I’m already finding harder than the others. It could be due to the distinct lack of sleep last night. What are your favourite self care activities to do? I need motivation to get off the sofa today #lockdown #lockdownblues

...everyone is rotting in their own houses and getting depression I’m not saying they’re less important but clearly this going in and out of lockdown isn’t helping anyone is it

this lockdown is starting to get to me – went off my food, crying more and just generally depressed loool

## Discussion

### Principal Findings

In this study, we identified and analyzed 286,902 geolocated tweets posted from users in the United Kingdom from July 23, 2020 to January 6, 2021 using a commercially available NLP platform. The findings showed that there was a fairly consistent trend in the volume of tweets over the study period, with spikes typically occurring during (or leading up to) a major change in social distancing measures in the United Kingdom. The NLP platform calculated an overall sentiment score of 50% indicating neutral sentiment across all tweets over the study period. Similar to volume, major fluctuations in sentiment appeared to coincide with major changes to lockdown rules.

Key topics of discussion that emerged consistently throughout the study period included (1) the impact that the pandemic and resulting lockdowns had been having on people’s mental health, both positive and negative; (2) fear and anxiety around the prospect of prolonged and subsequent lockdowns and how this might (or continue to) affect people’s mental health; and (3) anger and mistrust toward the government concerning a perceived lack of support for people struggling with their mental health.

Later in (and less consistently discussed throughout) the study period, other topics linked with mental health emerged, including sleep difficulties, increased alcohol intake, and anxieties concerning testing and the vaccine rollout.

Before the study, we anticipated that topics of discussion relating to mental health would be mostly underpinned with negative sentiment. It was therefore surprising that the findings of the topic analysis revealed higher levels of positive sentiment across posts associated with mental health. Consistently over the study period, people took to Twitter to share practical tips, strategies, and resources that could be used to support one’s mental health, and the platform was effective in clustering these types of posts with positive sentiment.

The viral spread of misinformation and “fake news” has represented a critical issue generating mass confusion, fear, and insecurity surrounding COVID-19 [19]. The WHO has repeatedly used the term “infodemic” to describe the sheer overabundance of misinformation being shared throughout the pandemic [20]. Our findings provide further example and insight into the rapid spread of health-related misinformation, particularly via channels of soft intelligence like social media. Specifically, in the case of this study, a copy-and-paste tweet campaign falsely claiming that suicide rates had increased by 200% since the first lockdown was shared widely by users. This particular cluster of tweets ranked as the topmost discussion topic during weeks 13 to 24 over the study period.

Overall, the results of this study demonstrate that using NLP to mine and analyze sources of soft intelligence (like Twitter) can yield useful health-related insights, which agencies, local leaders, and health care decision makers can potentially draw from. These findings contribute to a growing body of literature examining the value of this type of analyzed evidence and how it might support, link to, and (where appropriate) replace more traditional survey-based methods and data [15,21-24].

### Limitations

Several limitations can be attributed to this study. First, there are still considerable limitations concerning the reliability, accuracy, and transparency of the technologies in play. As an example, on examining the results of the NLP platform’s topic analysis, some of the tweets collated were not relevant to mental health (despite being identified as such). For example, tweets contained within clustered topics like “care homes” and “covid deaths” were expressing anger at the government, rather than negatively discussing mental health problems.

Some of the tweets included by the platform in its analysis were posted by businesses or charitable organizations, rather than members of the public. Such tweets, which often advertised local or national mental health services or shared self-improvement strategies, were typically classified by the platform as having positive sentiment. This created a large amount of background noise and skewed the overall sentiment toward positive. Further, tweets were not deduplicated by the platform, nor was there any formal analysis accounting for potential bot traffic. These factors will also have impacted the results.

In addition, despite the popularity of Twitter as a social networking tool, its users are not an accurate representation of the overall demographic of a population. Therefore, if we are to consider using Twitter (and similar resources) as a potential intelligence source, we must be mindful of bias concerning the key demographic information among its users (such as age, gender, and socioeconomic status).

There was also a number of limitations specific to the NLP platform that we selected. We had originally planned to run the topic analysis using the platform across all of the tweets as a single corpus. However, the platform was not able to process and analyze such a large volume of tweets in one go. Therefore, we had to split and run the topic analysis over 2 time periods (weeks 1 to 12 and weeks 13 to 24). Further, it was not possible to retrospectively search for and collect historic tweets, thus restricting possible options for analysis. Finally, although the broader methodologies that power the platform are touched on by its developers, the finer technical detail is not shared publicly due to commercial reasons.

### Conclusions

In this work, we analyzed a large collection of UK tweets relating to mental health during the COVID-19 pandemic to further explore the value of soft intelligence leveraged using NLP. Using a specialist, off-the-shelf, NLP platform, we collated a large corpus of tweets over a 24-week period and carried out various analyses to explore the volume, sentiment, and key trends and topics of discussion.

Our findings provide further evidence that this type of research is potentially a highly useful and efficient means to gain a rapid understanding of the key messages, concerns, and issues people are facing at scale. In the case of this reported study, we were able to draw insights into how the pandemic may be impacting people’s mental health and well-being by examining both the topic and sentiment specific to the UK population. This type of real-time analysis and intelligence may be particularly useful in helping shape rapid and reactive public health engagement and communication strategies during a health crises like COVID-19.

## Abbreviations

AI: artificial intelligence
ML: machine learning
NIHR: National Institute for Health Research
NLP: natural language processing
w/c: week commencing
WHO: World Health Organization

## References

[1] Wang C, Horby PW, Hayden FG, Gao GF (2020). A novel coronavirus outbreak of global health concern. Lancet.

[2] Cucinotta D, Vanelli M (2020). WHO declares COVID-19 a pandemic. Acta Biomed.

[3] (2020). Coronavirus: Two cases confirmed in UK. BBC News.

[4] Holmes EA, O'Connor RC, Perry VH, Tracey I, Wessely S, Arseneault L, Ballard C, Christensen H, Cohen Silver R, Everall I, Ford T, John A, Kabir T, King K, Madan I, Michie S, Przybylski AK, Shafran R, Sweeney A, Worthman CM, Yardley L, Cowan K, Cope C, Hotopf M, Bullmore E (2020). Multidisciplinary research priorities for the COVID-19 pandemic: a call for action for mental health science. Lancet Psychiatry.

[5] Lee J, Kim J, Hong YJ, Piao M, Byun A, Song H, Lee HS (2019). Health information technology trends in social media: using Twitter data. Healthc Inform Res.

[6] Chew C, Eysenbach G (2010). Pandemics in the age of Twitter: content analysis of Tweets during the 2009 H1N1 outbreak. PLoS One.

[7] Signorini A, Segre AM, Polgreen PM (2011). The use of Twitter to track levels of disease activity and public concern in the U.S. during the influenza A H1N1 pandemic. PLoS One.

[8] Odlum M, Yoon S (2015). What can we learn about the Ebola outbreak from tweets?. Am J Infect Control.

[9] Shin S, Seo D, An J, Kwak H, Kim S, Gwack J, Jo M (2016). High correlation of Middle East respiratory syndrome spread with Google search and Twitter trends in Korea. Sci Rep.

[10] Sinnenberg L, DiSilvestro CL, Mancheno C, Dailey K, Tufts C, Buttenheim AM, Barg F, Ungar L, Schwartz H, Brown D, Asch DA, Merchant RM (2016). Twitter as a potential data source for cardiovascular disease research. JAMA Cardiol.

[11] Tavoschi L, Quattrone F, D'Andrea E, Ducange P, Vabanesi M, Marcelloni F, Lopalco PL (2020). Twitter as a sentinel tool to monitor public opinion on vaccination: an opinion mining analysis from September 2016 to August 2017 in Italy. Hum Vaccin Immunother.

[12] Hussain A, Sheikh A (2021). Opportunities for Artificial Intelligence–Enabled Social Media Analysis of Public Attitudes Toward Covid-19 Vaccines. NEJM Catalyst: Innovations in Care Delivery.

[13] Wordnerds.

[14] Wordnerds.

[15] Twitter API. Twitter Developer.

[16] (2019). A Twitter study of the UK Rail Industry. Wordnerds.

[17] Appleby L, Richards N, Ibrahim S, Turnbull P, Rodway C, Kapur N (2021). Suicide in England in the COVID-19 pandemic: Early observational data from real time surveillance. Lancet Reg Health Eur.

[18] Krishna R There is no evidence that suicides have increased 200% under lockdown. Full Fact.

[19] Freiling I, Krause NM, Scheufele DA, Brossard D (2021). Believing and sharing misinformation, fact-checks, and accurate information on social media: The role of anxiety during COVID-19. New Media & Society.

[20] Eysenbach G (2020). How to fight an infodemic: the four pillars of infodemic management. J Med Internet Res.

[21] Al Baghal T, Sloan L, Jessop C, Williams ML, Burnap P (2019). Linking Twitter and survey data: the impact of survey mode and demographics on consent rates across three UK studies. Social Science Computer Review.

[22] Hung M, Lauren E, Hon ES, Birmingham WC, Xu J, Su S, Hon SD, Park J, Dang P, Lipsky MS (2020). Social network analysis of COVID-19 sentiments: application of artificial intelligence. J Med Internet Res.

[23] Lyu JC, Han EL, Luli GK (2021). COVID-19 vaccine-related discussion on Twitter: topic modeling and sentiment analysis. J Med Internet Res.

[24] Valdez D, Ten Thij M, Bathina K, Rutter LA, Bollen J (2020). Social media insights into US mental health during the COVID-19 pandemic: longitudinal analysis of Twitter data. J Med Internet Res.

